# Anxiety and depression in metabolic-associated steatotic liver disease: relation with socio-demographic features and liver disease severity

**DOI:** 10.1007/s00592-024-02287-0

**Published:** 2024-04-29

**Authors:** Lucia Brodosi, Michele Stecchi, Alessandra Musio, Matilde Bazzocchi, Eleonora Risi, Francesca Marchignoli, Giulio Marchesini, Maria Letizia Petroni

**Affiliations:** 1 Department of Medical and Surgical Sciences, University of Bologna, Bologna, Italy; 2 Clinical Nutrition and Metabolism Unit, IRCCS-Azienda Ospedaliero-Universitaria Di Bologna, Bologna, Italy; 3 Alma Mater University, Bologna, Italy

**Keywords:** Nonalcoholic fatty liver disease, Metabolic dysfunction-associated steatotic liver disease, Vibration-controlled transient elastography, Liver fibrosis, Distress, Biomarkers, Imaging

## Abstract

**Purpose:**

We aimed to evaluate the prevalence of anxiety and depression traits in Italian patients with metabolic dysfunction-associated steatotic liver disease (MASLD), and the possible relation with the severity of liver disease.

**Methods:**

Demographic, anthropometric, clinical and laboratory parameters were collected in patients referred to a metabolic unit for a comprehensive evaluation of possible liver disease. Hepatic steatosis and fibrosis were evaluated by surrogate biomarkers. Imaging (controlled attenuation parameter-CAP and vibration-controlled transient elastography-VCTE). Beck depression inventory (BDI) and state-trait anxiety inventory-Y (STAI-Y) were used to define depressive/anxiety states; calorie intake and lifestyle were self-assessed by questionnaires.

**Results:**

The whole sample comprised 286 patients (61.9% females; mean age 52.0 years; BMI, 34.6 kg/m^2^); 223 fulfilled MASLD criteria. BDI and trait anxiety scores were lower in the MASLD cohort, and the prevalence of both moderate/severe depression and severe trait anxiety was reduced compared with non-MASLD cases, despite VCTE-diagnosed fibrosis F3–F4 present in over 15% of cases. However, after correction for demographic and anthropometric confounders, MASLD was not associated with a lower risk of moderate/severe depression or severe anxiety trait (odds ratio, 0.34; 95% confidence interval, 0.12–1.01 and 0.79, 0.27–2.34). Additional adjustment for the severity of fibrosis did not change the results. No differences in state anxiety were observed.

**Conclusion:**

The risk of anxiety and depression in MASLD is not different from that generated by diabetes and obesity per se*.* MASLD patients do not perceive liver disease as a specific source of psychological distress, possibly as a consequence of the unawareness of progressive liver disease.

## Introduction

The term steatotic liver disease (SLD) has been recently chosen as the overarching definition of hepatic steatosis identified by imaging or biopsy [1]. It includes metabolic dysfunction-associated steatotic liver disease (MASLD), formerly known as nonalcoholic fatty liver disease (NAFLD), the most common form of liver disease worldwide. A systematic review and meta-analysis reported an increase from 25.3% in the period 1990–2006 to 38% in more recent epidemiological surveys (2016–2019)[2], and MASLD is now the second cause of liver transplantation in the United States and Europe, and the first in women [3, 4].

Despite massive efforts by pharmaceutical companies and multiple lines of research, lifestyle intervention and weight loss are the only strategies for MASLD treatment approved by international guidelines to reduce disease progression [5, 6]. Unfortunately, only few patients achieve and maintain the desired lifestyle modifications in diet and physical activity associated with long-term weight loss (10% or more), reported to improve disease outcome [7].

One of the factors influencing attitudes towards diet and exercise is mental health. The presence of anxiety or depression can affect motivation and compliance to treatment in chronic diseases [8]. In anxiety states, food intake may be a way of coping with stress, while in depression it may help experiment positive emotions [9, 10], and adult women with positive screening for anxiety or depression have higher scores in uncontrolled eating and emotional eating [11]. Depression is a common public health problem, affecting 12.9% of individuals globally, with differences among countries, gender and other socioeconomic factors [12]. Also generalized anxiety disorder is very common in both community and clinical settings. Worldwide, estimates of lifetime and 12-month prevalence are 3.7% and 1.8%, respectively [13], but the prevalence of less severe disorders may be much higher, particularly in the presence of comorbid conditions.

Only a few studies are available on the prevalence of anxiety/depression in the MASLD population, with conflicting results. A meta-analysis by Xiao et al. [14], involving over two-million patients, found that NAFLD was associated with an increased risk of depression (OR: 1.29, CI: 1.02–1.64, *p* = 0.03), whereas a Brazilian study reported an inverse association with anxiety [15]. Weinstein et al. reported that individuals with NAFLD and depression were more likely to be physically inactive (60.7%) compared to NAFLD individuals without depression (33.3%) [16], and the presence of depression was associated with worse treatment outcomes [17]. Finally, an association was reported between depression and all-cause [18, 19] and cancer-related mortality [18], cardiovascular diseases [18] and stroke [18] in NAFLD.

Providing psychological support to frail patients is mandatory to improve the effectiveness of lifestyle intervention. We aimed to define the prevalence of anxiety and depression traits in consecutive Italian patients with MASLD, and the possible relation with liver disease severity. This strategy might help define the importance of psychologists in the team building inside the MASLD treatment units, as prerequisite to adapt lifestyle programs to the possible presence of psychologic burden.

## Material and methods

### Patients

The present study is part of the ongoing program "One day screening of NASH", a study started in June 2021 at the IRCCS AOUBO (University of Bologna) aimed at evaluating the presence of hepatic steatosis and the risk of liver fibrosis, measured with non-invasive methods, in a population with suspected metabolic syndrome and referred to our Institution by general practitioners or specialists of other clinical areas without a history, or signs and symptoms of advanced liver disease. According to the program, subjects entering the program are visited by a specialist in metabolic disorders and are screened for steatotic liver disease (SLD) by non-invasive methods. Inclusion criteria were informed consent signed before any trial-related activities, age ≥ 18 years, presence of at least one feature of metabolic syndrome; exclusion criteria were alcohol intake  ≥ 20 g/day (women and 30g/day (men) and active hepatitis B and C virus infection.

The study was performed in accordance with the ethical standards as laid down in the 1964 Declaration of Helsinki and its later amendments. It was approved by the ethical committee of Area Vasta Emilia Centro (Study 110/2021/Sper/AOUBo); all subjects signed the informed consent to participate in the study and to report publication.

### Methods

All subjects were tested when first seen at our Institution, before any intervention, using a protocol previously described [20]. The visit included questions on lifestyle habits, including self-evaluated food intake and physical activity (graded from 1 [much lower-than-normal, sedentary] to 5 [much higher-than-normal, very physically active] compared to relatives and friends), smoking and alcohol use. Cases reporting alcohol use above 14 units/week in women and 21 units/week in men were excluded from the analysis. The diagnosis of Metabolic Syndrome was based on the redefinition of the National Cholesterol Education Program—Adult Treatment Panel III (NCEP-ATPIII) [21], which requires the presence of at least three of the following five criteria: (a) waist circumference ≥ 94 cm in men and ≥ 80 in women; (b) triglycerides ≥ 150 mg/dL or ongoing lipid-lowering therapy; (c) HDL cholesterol ≤ 40 mg/dL in men and ≤ 50 in women; (d) systolic blood pressure ≥ 130 mmHg and diastolic pressure ≥ 85 or current antihypertensive therapy; (e) fasting blood glucose ≥ 100 mg/dL or diagnosis of diabetes mellitus.

Fasting blood samples were drawn for the determination of a panel of laboratory parameters, if not available within 90 days prior to the visit. All laboratory tests were performed by the Metropolitan laboratory Service of Bologna. The upper limits of alanine aminotransferases (ALT) were set at 30 U/L in men and 19 U/L in women [22]. The Fatty Liver index (FLI) was calculated as marker of steatosis; based on the FLI score, patients were divided into three categories: low risk of steatosis (FLI < 30), intermediate risk of steatosis (FLI between 30 and 60) and high risk of steatosis (FLI > 60) [23]. Similarly, the presence of advanced fibrosis was estimated by the Fibrosis-4 index (Fib-4) [24]. Fib-4 values < 1.30 exclude advanced fibrosis, values > 2.67 are at high risk of advanced fibrosis, values between 1.30 and 2.67 are in the indeterminate range [25].

Finally, all patients were tested by vibration-controlled transient elastography (VCTE) using FibroScan™ (Echosense, Paris, France). Both liver stiffness (marker of fibrosis) and the Controlled Attenuation Parameter (CAP), marker of steatosis, were determined [26]. M or XL probes were used according to standard protocols. The probes undergo calibration every six months. The test was considered reliable if ten valid measurements were achieved with a success rate above 60% and the ratio between the interquartile range and the median was < 0.3. The stiffness cut-offs indicative of significant fibrosis (F2), advanced fibrosis (F3) and cirrhosis (F4) were 7.0 kPa, 8.7 kPa and 10.3 kPa, respectively. The CAP score diagnostic cut-offs were set at 248 dB/m, 260 dB/m and 280 dB/m, respectively, for grade 1 (S1), grade 2 (S2) and grade 3 (S3) steatosis.

### Questionnaires

*Depression*—The Beck Depression Inventory (BDI) was used to assess the presence and severity of self-reported depression levels. It consists of 21 items each rated from 0 (no symptom), to 3 (severe symptom). The BDI had an internal consistency of 0.81 and 0.85 in the sample with and without obesity, respectively, a reasonably good test–retest reliability, and good criterion validity [27]. Cutoff values for total BDI scores were used for descriptive presentation. The responses were graded according to the following cut-offs: normal, 0–9; mild depressive symptoms, 10–15; moderate depressive symptoms, 16–22; and severe depressive symptoms, 23–63 [28, 29]. Finally, it has been validated in the Italian version [30].

*Anxiety*—The State Trait Anxiety Inventory-Y (STAI-Y) [31] was used for the assessment of anxious symptomatology. It consists of two sub-scales of 20 items each: state anxiety (S-STAI-Y), the respondent’s level of anxiety at the present time, and trait anxiety (T-STAI-Y), the usual level of the respondent’s anxiety in his/her everyday life. Sample items for the STAI-Y are “I am tense” for state anxiety, and “I am a steady person” for trait anxiety. For both STAI-Y questionnaire, item scores vary from 0 to 4, with higher scores indicating higher levels of anxious symptoms (No or low anxiety, 20–37; moderate anxiety, 38–44; high anxiety, 45–80). The S-STAI-Y had an internal consistency of 0.80 and 0.84, while the T-STAI-Y of 0.80 and 0.82 in the sample with and without obesity, respectively. Finally, the reliability of both STAI-Y Forms in their Italian version has been validated [32].

*Dietary intake*—*“Quanto Mangio Veramente”* (How much do I really eat; QMV, 20 items). The questionnaire semi-quantitatively estimates calorie intake on the basis of the habitual weekly consumption and portion size (on a 5-point Likert scale) of 18 items referred to habitual food intake, and a final item on the number of meals not consumed at home during the week [33]. To help subjects with the determination of portion size, pictures are presented to visually explain what is considered small-sized, medium-sized, or large-sized, whereas a few questions specifically investigate the number of specific items consumed during the week, if any (e.g., number of sugar cubes or sugar coffee-spoons, candies, chocolate tablets). Each portion size is given an estimate (value) of its energy content (as multiple of 50 kcal, to simplify calculations). The in-house developed questionnaire has been validated vs. dietary interview carried out by an expert dietitian and has been extensively used since 2006 by specialists and by general physicians in the area of Bologna [34].

### Statistical analysis

The statistical analysis was carried out by means of StatView 5.0 program (SAS Institute, Inc., Cary, NC.) and SPSS for Windows v.21 (SPSS Inc., Chicago, IL, USA), on the whole population and separately in the two cohorts. Descriptive statistics included mean, standard deviation (SD), median (interquartile range) for non-Gaussian distributed scores, and percentage for categorical variables. Comparison between different groups were carried out by Student t test for unpaired data or chi-square values, as appropriate. Non parametric analyses (Mann–Whitney or Wilcoxon test) were also performed. Correlation analysis and/or linear and logistic regression analysis (odds ratio [OR] and 95% confidence interval [95% CI]) were used to test the association between BDI and STAI scores or risk of depression and anxiety with clinical data, with/without adjustment for confounders. For all comparisons, *P* values < 0.05 were considered statistically significant.

## Results

### Demographic, anthropometric and clinical data

In the period between June 2021 and December 2023 the protocol involved 286 subjects, and CAP identified the presence of steatosis of variable severity in 223. Their clinical and laboratory data are reported in Table 1, divided according to the presence of MASLD. Notably, subjects with MASLD (n = 223) were characterized by older age and lower education, and were more frequently males. Their lifestyle was not remarkably different from that reported from the non-MASLD group (n = 63), also in terms of food intake and engagement in physical activity. Their BMI was however nearly six-point higher, with a totally different distribution across the obesity grades, despite very much similar reported BMI at age 20, expression of a remarkable weight increase, which had also occurred in the past 12 months in both cohorts. The MASLD cohort was also characterized by a higher prevalence of diabetes, hypertension and metabolic syndrome. Finally, their liver enzymes were more commonly elevated (only 30.8% had normal ALT vs. 55.7% in non-MASLD). FLI values were consistent with the presence of steatosis indicated by CAP (coefficient of correlation between CAP and FLI: R^2^, 0.272; *P* < 0.001), and this was also the case for FIB-4 and vibration-controlled transient elastography (VCTE) (coefficient of correlation between liver stiffness and FIB-4: R^2^, 0.406; *P* < 0.001), with a risk of advanced fibrosis detected also in a minority of non-MASLD cases.

**Table 1:** Demographic, anthropometric, clinical and laboratory data of patients with/without steatotic liver disease (SLD) involved in the study

	All cases (n = 286)	MASLD (n = 223)	No-MASLD (n = 63)	*P* value*
*Demography*				
Age (years)	52.0 ± 12.5	52.9 ± 12.3	48.9 ± 12.6	0.026
Female sex (%)	61.9	56.9	79.4	0.023
Civil status (single/married/ widowed) (%)	35.0/61.3/3.7	34.4/61.3/4.3	36.4/61.4/2.3	0.897
Education (primary/secondary/ vocational/degree) (%)	1.5/14.6/49.6/34.2	2.2/19.4/53.8/24.7	–/4.5/40.9/54.5	0.014
*Self-assessed lifestyle habits*				
Food intake (kcal/day)^$^	1996 ± 637	1982 ± 602	2027 ± 637	0.700
Food intake (1–5) (%)°	1.5/14.1/41.5/37.3/6.6	2.2/11.0/42.9/37.4/6.6	–/20.5/38.6/31.8/9.1	0.480
Physical activity (1–5) (%)^	20.7/40.7/24.4/12.6/1.5	17.9/47.4/23.1/11.5/–	24.6/32.7/26.3/14.0/3.5	0.217
Alcohol intake (safe limits/abstainer/ex) (%)	46.7/47.4/5.9	48.7/48.7/2.6	43.9/45.6/10.5	0.161
Smoking (yes/no/ex) (%)	26.3/45.9/27.8	31.5/46.1/22.4	15.9/45.5/38.6	0.065
*Clinical data*				
BMI (kg/m^2^)	34.6 ± 5.4	36.0 ± 5.2	29.8 ± 2.5	< 0.001
Overweight/Obesity I, II, III (%)	11.5/53.8/18.6/16.1	2.2/53.4/23.8/20.6	37.7/56.5/5.8/–	< 0.001
∆ BMI in the last year (%)	3.3 ± 8.0	3.3 ± 7.3	3.3 ± 9.4	0.987
BMI at age 20 (kg/m^2^)	23.8 ± 4.6	24.4 ± 5.0	22.7 ± 3.2	0.053
Waist circumference (cm)	107.3 ± 13.7	111.2 ± 12.4	95.0 ± 9.5	< 0.001
Diabetes (%)	26.2	30.1	12.1	0.038
Blood glucose (mg/dL)	99.2 ± 19.2	100.6 ± 19.0	94.2 ± 19.3	0.020
HbA1c (mmol/mol)	39.8 ± 7.9	40.2 ± 8.1	38.5 ± 7.5	0.337
Triglycerides (mg/dL)	135.9 ± 71.7	146.2 ± 75.2	99.7 ± 40.8	< 0.001
HDL-cholesterol (mg/dL)	52.6 ± 11.3	51.7 ± 11.0	56.0 ± 11.8	0.006
Hypertension (%)	32.9	36.7	19.0	0.006
Systolic blood pressure (mmHg)	125.0 ± 15.7	127.0 ± 15.1	117.5 ± 15.9	< 0.001
Diastolic blood pressure (mm/Hg)	76.9 ± 10.4	78.1 ± 10.1	72.9 ± 10.4	< 0.001
Metabolic syndrome (%)	41.3	48.9	14.3	< 0.001
No of features (1,2,3,4,5) (%)	32.9/37.1/22.7/6.3/1.0	27.4/35.9/27.4/8.1/1.3	52.3/41.3/6.3/–/–	< 0.001
Alanine aminotransferases (U/L)	33.5 ± 24.9	37.3 ± 27.1	21.7 ± 9.8	< 0.001
Normal ALT (%)^#^	37.0	30.8	55.7	0.001
Aspartate aminotransferases (U/L)	28.5 ± 17.6	30.3 ± 19.4	22.5 ± 7.2	0.003
Gamma-GT (U/L)	37.7 ± 30.0	42.4 ± 32.3	21.0 ± 7.3	< 0.001
*Biomarkers and Imaging*				
Fatty liver index (%)	77.8 ± 20.8	87.1 ± 9.8	45.0 ± 15.2	< 0.001
< 30/31–60/ > 60 (%)	3.5/14.7/81.8	- /0.9/99.1	15.9/63.5/20.6	< 0.001
Fibrosis-4 score	1.23 ± 2.05	1.30 ± 2.33	0.99 ± 0.54	0.341
< 1.30/1.31–2.67/ > 2.67 (%)	71.6/22.3/6.1	70.9/21.7/7.4	74.1/24.1/1.8	0.323
Controlled attenuation parameter (dB/m)	270.7 ± 51.8	291.2 ± 45.3	220.7 ± 26.1	< 0.001
S0/S1/S2/S3 (%)	39.7/7.3/13.2/39.8	–/12.1/22.0/65.9	100.0/–/–/–	< 0.001
Liver stiffness (VCTE) (kPa)	6.34 ± 5.29	6.94 ± 5.83	4.21 ± 1.18	< 0.001
F0–F1/F2/F3/F4 (%)	77.3/7.7/6.6/8.4	71.7/9.0/8.5/10.8	96.8/3.2/–/–	< 0.001

Data are expressed as mean ± standard deviation or as prevalence, as appropriate

^*^Student t test, Chi-square test, or Fisher’s exact test, as appropriate. For multiple categorical variables, P value refers to overall differences between groups (Chi-square test)

°Self-graded from 1 to 5 as: very low, low, normal, higher-than-normal, much higher-than-normal

^Self-graded from 1 to 5 as: very sedentary, sedentary, normal, moderately active, very active

^#^ALT, alanine aminotransferase: normal values, < 31 U/L in men, 20 U/L in women

### Depression scores

**Fig. 1:**
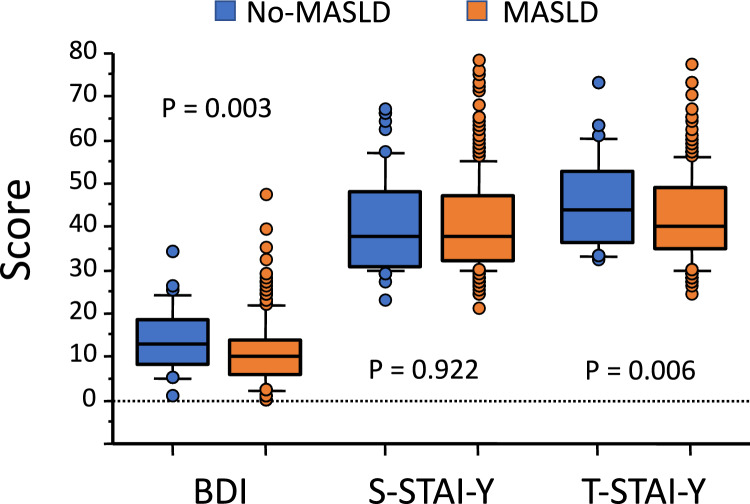
Scores of depressive mood (beck depression inventory [BDI]) and anxiety (S- and T-state and trait depression inventory form-Y [S- and T-STAI-Y]) in the population with/without MASLD. In this box and whiskers plot, the horizontal lines correspond to medians, the boxes cover the 25°-75° area and the whiskers extend to 5–95° of variance. The circles represent cases exceeding the 95% confidence intervals. No difference between paired plots were observed

**Fig. 2:**
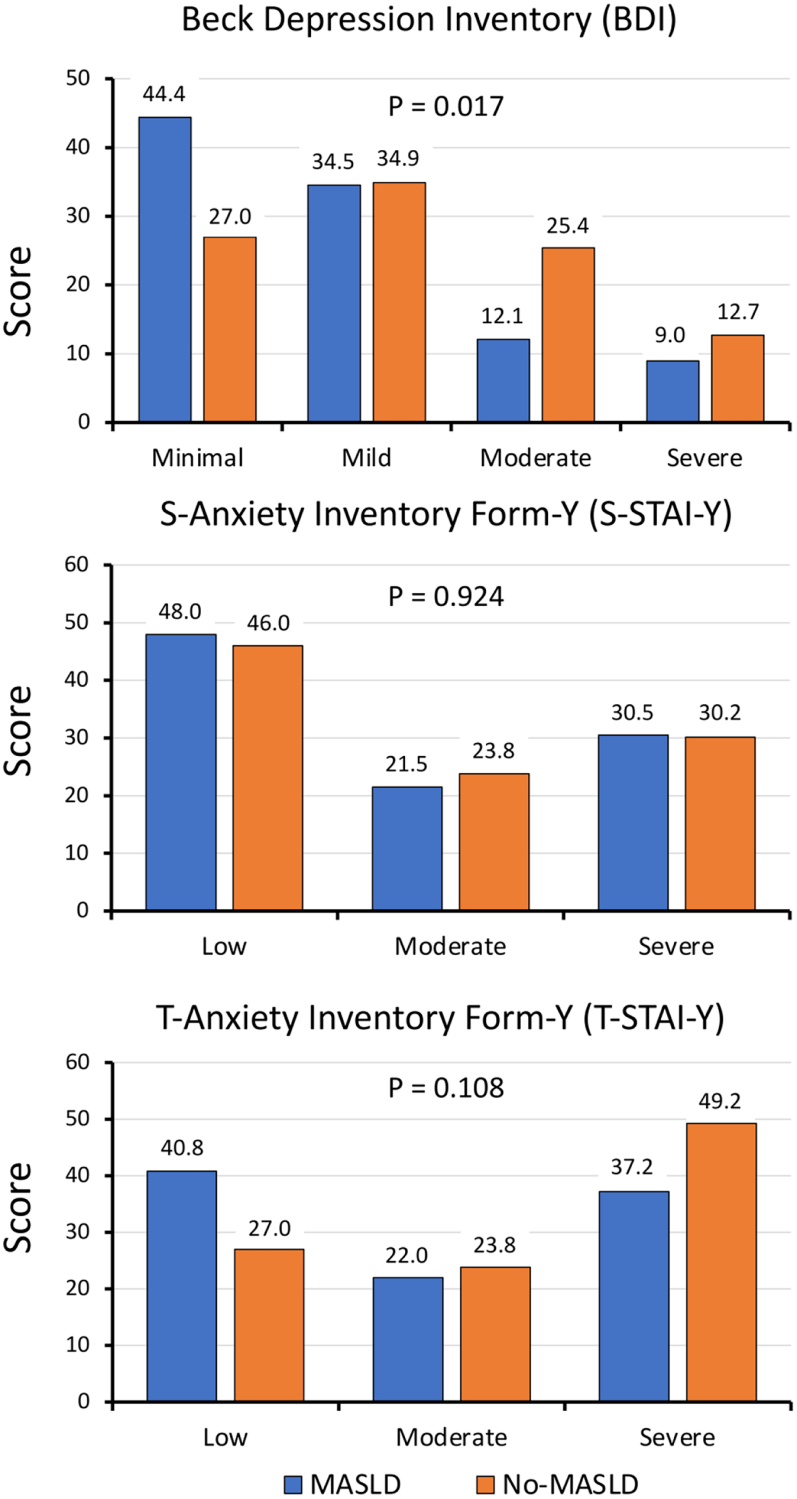
Prevalence of depression and anxiety scores according to categories of disease severity. No difference in score distribution was observed between No-MASLD and MASLD cohorts (Chi-square test)

In the whole population, BDI scores ranged from 0 to 47; they were moderately higher in women (n = 177; median, 12; interquartile range [IQR], 9) compared to men (n = 109; 10 [9]; *P* = 0.006), and in subjects aged ≤ 60 years (n = 205; median 11, [9]) vs. older patients (n = 81; 9, [8.25]; *P* = 0.004) and the differences were maintained in the MASLD cohort (Table 2). The values were more dispersed and significantly lower in the presence of MASLD (10 [8] vs. 13 [10.5] in non-MASLD; *P* = 0.003) (Fig. 1); also when classified according to categories of depression severity, many more cases with severe depression were observed in the non-MASLD cohort (*P* = 0.021) (Fig. 2), with a prevalence of moderate/severe depression nearly doubled in non-MASLD (38.1% vs. 21.1% in MASLD; Fisher’s exact test, *P* = 0.008). In a logistic regression analysis, the presence of MASLD was associated with a lower risk of moderate/severe depression (OR, 0.43; 95% CI, 0.24–0.79; *P* = 0.006), but the association was cancelled by adjustment for demographic (age, sex, civil status and education) and anthropometric data (BMI) (OR, 0.34; 95% CI, 0.12–1.01; *P* = 0.052). Additional correction for the severity of liver fibrosis, as measured by VCTE, did not change the results. Notably, the prevalence of moderate/severe depression increased with increasing BMI and was as high as 37% in subjects with grade 3 obesity vs. 22% in lower obesity grades (*P* = 0.042).

**Table 2:** Scores of beck depression inventory (BDI) and state and trait anxiety inventory form-Y (S-STAI-Y and T-STAI-Y) in subjects with/without MASLD, grouped according to demographic, anthropometric and clinical characteristics (median [interquartile range—IQR])

Questionnaire	MASLD (223)		*P* value	Non-MASLD (63)		*P* value	*P* value	*P* value
	(a)	(b)	(a) vs. (b)	(c)	(d)	(c) vs. (d)	(a) vs. (c)	(b) vs. (d)
Beck depression inventory (BDI)	Men (n = 96)	Women (n = 127)	**0.004**	Men (n = 13)	Women (n = 50)	0.206	**0.004**	0.278
	9 [7.5]	12 [7.75]		15 [11]	13 [10]			
	Age ≤ 60 (n = 152)	Age > 60 (n = 71)	**0.016**	Age ≤ 60 (n = 53)	Age > 60 (n = 10)	0.553	**0.037**	0.126
	11 [8]	9 [7]		13 [9.25]	13 [7]			
	F0–F2 (n = 180)	F3–F4 (n = 43)	0.078	F0–F2 (n = 63)	F3–F4 (n = 0)	–	**0.001**	–
	10 [8]	12 [7.75]		13 [10.5]	–			
State anxiety inventory form-Y (S-STAI-Y)	Men (n = 96)	Women (n = 127)	**0.001**	Men (n = 13)	Women (n = 50)			
	36 [9]	40 [17.75]		33 [13.5]	39.5 [19]	0.126	0.364	0.634
	Age ≤ 60 (n = 152)	Age > 60 (n = 71)	**0.026**	Age ≤ 60 (n = 53)	Age > 60 (n = 10)	0.547	0.704	0.931
	38.5 [15.5]	36 [9]		38 [20]	34.5 [14]			
	F0–F2 (n = 180)	F3–F4 (n = 43)	0.392	F0–F2 (n = 63)	F3–F4 (n = 0)	–	0.934	–
	38 [16]	43 [15.75]		38 [17]	–			
Trait anxiety inventory form-Y (T-STAI-Y)	Men (n = 96)	Women (n = 127)	** < 0.001**	Men (n = 13)	Women (n = 50)	0.405	**0.003**	0.440
	37 [11]	43 [14]		48 [14.5]	44 [17]			
	Age ≤ 60 (n = 152)	Age > 60 (n = 71)	**0.028**	Age ≤ 60 (n = 53)	Age > 60 (n = 10)	**0.017**	0.298	** < 0.001**
	38 [13.75]	41.5 [14.5]		43 [16.25]	51.5 [12]			
	F0–F2 (n = 180)	F3–F4 (n = 43)	0.157	F0–F2 (n = 63)	F3–F4 (n = 0)	–	**0.003**	–
	39 [13]	43 [15.75]		44 [16.75]	–			

Data are analyzed by Mann–Whitney U test

Significant values are printed in bold characters

In MASLD higher depression scores were demonstrated in the presence of advanced liver fibrosis, measured by VCTE (fibrosis F3–F4, median 12 (IQR, 7.75) vs. 10 [8] in fibrosis F0–F2; *P* = 0.078), not when fibrosis was scored by the surrogate biomarker FIB-4 (Rule-out fibrosis, 11 [8.5]; Indeterminate, 10 [5]; Rule-in Fibrosis, 9 [14]; *P* = 0.645).

### Anxiety scores

Notably, State and Trait anxiety scores were correlated with BDI scores in the whole cohort (r = 0.509 and r = 0.640, respectively), as were S-STAI-Y vs. T-STAI-Y (r = 0.694; all, *P* < 0.001) (Fig. 1). Both S-STAI-Y and T-STAI-Y scores were significantly higher in women (median 40 (18) and 41 (15) vs. 36 (9) and 37 (12); *P* < 0.001 for both), and differences were maintained in the MASLD cohort, where scores were more dispersed (Table 1 and Fig. 1). State anxiety scores were again higher in young persons in MASLD, whereas trait scores were higher in older subjects in both cohorts. Finally, severe anxiety scores were more common in the non-MASLD group (without statistically significant differences vs. MASLD cohort) (Fig. 2). In the MASLD cohort, the presence of more severe VCTE-diagnosed fibrosis was not associated with higher state and trait anxiety scores. In logistic regression analyses, the presence of MASLD was not associated with either mild or severe anxiety states, but it was again negatively associated with a severe anxiety trait (severe T-STAI-Y, OR, 0.50; 95% CI, 0.26–0.97; *P* = 0.040). Also in this case, the risk was no longer significant after adjustment for demographic and anthropometric confounders (OR, 0.79; 95%CI, 0.27–2.34), without any effect of the presence of advanced fibrosis.

### Relation between depression/anxiety and body weight

No association was demonstrated between BDI score and BMI (R^2^ = 0.005; *P* = 0.249) or between BDI class and obesity grades (*P* = 0.127), but severe depression was associated with 5% or more weight loss or weight gain in the previous year (*P* = 0.038), not with self-assessed food intake (*P* = 0.132) in the whole cohort, as well as in MASLD.

S-STAI-Y was associated with BMI in the whole cohort (R^2^ = 0.017; *P* = 0.026), and the correlation was maintained in MASLD (R^2^ = 0.029; *P* = 0.010), but was not associated with recent weight change.

No correlation was observed between T-STAI-Y score or severity and BMI, BMI change and obesity grades.

## Discussion

The study fails to identify significant states of anxiety and depression specifically associated with the presence of MASLD in our population screened for the presence of hepatic fat as suggested by guidelines in subjects with features of the metabolic syndrome. Compared to subjects without biochemical and imaging evidence of steatosis, they do not score worse in validated questionnaires of depression and anxiety, after correction for confounders. The most important factors increasing the risk of anxiety and depression remain younger age and female sex.

The relation between obesity and depression in the community is a matter of debate. In a systematic review and meta-analysis involving over 16,000 older adults (≥ 60 years) in 19 studies, the presence of overweight and obesity were associated with lower odds of depression, confirming the hypothesis of “jolly fat” [35]. This is probably not the case for younger subjects. A review article concluded that increasing adiposity from childhood to adulthood, as was probably the case for the majority of younger individuals enrolled in the present study considering the large weight gain from age 20, was associated with an increased risk of depression, particularly in women, not in men [36]. This was confirmed in our setting, where the higher BDI scores observed in individuals aged below 60 in comparison to younger patients were mostly due to the difference observed in women.

The negative association of MASLD with depression and trait anxiety, when compared with non-MASLD cohort, should be interpreted with caution. As seen, it largely stems from the older age and the higher prevalence of men in MASLD (two factors associated with a reduced risk of depression), and the difference is no longer significant after adjustment for confounders, including differences in education, largely reflected in different working activities. Indeed, other studies reported positive associations between depression/anxiety and NAFLD. In a retrospective cross-sectional study involving 25,333 subjects, NAFLD was significantly associated with depression (_adjusted_OR, 1.43; 95% CI, 1.14–1.80; *P* = 0.002), while severe NAFLD was associated with state and trait anxiety (_adjusted_OR, 1.84; 95% CI, 1.01–3.37; *P* = 0.047 and _adjusted_OR 2.45; 95% CI, 1.08–4.85; *P* = 0.018, respectively) in women [37]. Similarly, in a cross-sectional study conducted on 3,327 individuals from National Health and Nutrition Examination Survey, the prevalence of depression, assessed through the Patient Health Questionnaire (PHQ-9), was higher among individuals with MAFLD or significant fibrosis than among those without [38]. In a Brazilian cross-sectional study including 7,241 participants evaluated by 21-item Beck Anxiety Inventory, Patient Health Questionnaire-9, and K6 distress scale), NAFLD was inversely associated with anxiety and positively associated with depression. Finally, in a 10-year longitudinal study comparing 19,871 patients with NAFLD to 19,871 matched controls, the hazard ratio for the incidence of depression was 1.21 (*P* < 0.001) and that for the incidence of anxiety was 1.23 (*P* < 0.001) [39].

We could not find any reference value for anxiety/depressive mood in the Italian population, measured by the questionnaires used in the present study. The large meta-analysis by Lim et al. across different countries provides cross-sectional, one-year and lifetime prevalence values of depression of 12.9%, 7.2% and 10.8% respectively [12], which are in keeping with the rate of severe depression in our study. The same report indicates that the prevalence is higher in women (14.4%), in countries with a medium human development index (HDI) (29.2%), and when tested by self-reported instruments (17.3%). In general, the prevalence of depressive/anxiety symptoms mixed as bipolar disorder [40], is higher than normal in the presence of obesity and other features of the metabolic syndrome, where a bi-directional association and the existence of a “metabolic-mood syndrome” was also postulated [41]. It is noteworthy that the non-MASLD cohort showed an unexpectedly high prevalence of anxiety-depression at questionnaires, despite their lower obesity rates. Whether these less severe patients represent a cohort representative of the general population or a cohort specifically seeking medical referral for psychosomatic distress remains to be defined.

State and trait anxiety scores were partly different between MASLD and non-MASLD cases. S-STAI-Y provides a measure of the psychological reactivity directly related to a specific situation and in a specific moment, whereas T-STA-Y reflects a particular trait of personality. For both cohorts, differences were detected in relation to gender and age, but the trait scores were lower in MASLD, in keeping with the lower scores of BDI, and confirming a different grade of psychological distress.

Within this complex scenario, mediated by bio-psychosocial factors, three additional components should be considered. Firstly, treatment of depression, per se, is likely to increase body weight [42], thus contributing to liver fat accumulation. The issue of psychotropic drug use was not systematically assessed in our analysis; individuals referred to our institution were free-living and limited data collected during triage do not support this possibility. However, this remains a limitation of the study, that should be more extensively investigated. Second, we did not find an association between recent weight change and anxiety or depressive categories. Significant weight loss and decreased appetite are included as diagnostic criteria for major depression in DSM-5-TR, but this condition was exceedingly rare in our setting. Third, depression and eating styles are systematically associated with dietary intake, namely lower adherence to the principles of Mediterranean diet and a risk of binge eating disorders (BED), but a previous study failed to detect an increase in BED prevalence in our population [20].

In conclusion, the presence of MASLD, largely unappreciated by patients when first visited in our unit, is not perceived as an additional source of psychological distress. The general unawareness of the risks associated with MASLD has been largely documented in the literature [43], and is a matter of concern for dedicated healthcare professionals, requiring intensive approach [44] and prompting the publication of dedicated guidelines for patients [45] and a global priority agenda [46]. BDI scores were in the range indicative of severe depressive state in over 18% of MASLD cases with fibrosis F3–F4 at VCTE—values diagnostic for cirrhosis in over 10% of cases –, but depressive mood remained much more common and predictive of non-MASLD. As supported by the high prevalence of depression in individuals with more severe obesity grades, this conclusion suggests that obesity per se probably remains the main clinical factor with an impact on mood for the majority of MASLD patients—and also for their general practitioners who refer patients to specialized centers on the basis of obesity grades, not considering their liver disease. Italian MASLD guidelines have been shared by the Liver, Diabetes and Obesity associations to facilitate screening of patients at risk of advanced MASLD on the basis of non-invasive biomarkers [47], following the evidence of poor preparedness of the healthcare network [48]. The evidence that metabolic liver disease generates a lower impact on anxiety/depression than that provided by the sole presence of obesity means that we have a long way to go before MASLD receives proper referral and treatment, but specialized centers should be prepared to provide adequate psychological support and to prepare specific programs for MASLD individuals with the most severe grades of obesity.

## Abbreviations

ALT: Alanine aminotransferases
BDI: Beck depression Inventory
BMI: Body mass index
CAP: Controlled attenuation parameter
DSM-5-TR: The diagnostic and statistical manual of mental disorders, fifth edition, text revision
FIB-4: Fibrosis-4 index
EE: Emotional eating
F0–F1: Absent or low liver fibrosis at VCTE
F2: Significant liver fibrosis at VCTE
F3: Advanced liver fibrosis at VCTE
F4: Liver cirrhosis at VCTE
FLI: Fatty liver index
MASLD: Metabolic dysfunction-associated steatotic liver disease
NAFLD: Nonalcoholic fatty liver disease
NASH: Nonalcoholic steatohepatitis
QMV: “*Quanto Mangio Veramente*” (How much do I really eat) questionnaire
SLD: Steatotic liver disease
STAI-Y: State-trait anxiety inventory-Y forms
VCTE: Vibration-controlled transient elastography

## References

[1] Rinella ME, Lazarus JV, Ratziu V, Francque SM, Sanyal AJ, Kanwal F et al (2023) A multisociety Delphi consensus statement on new fatty liver disease nomenclature. Hepatology 78:1966–1986. 10.1097/HEP.0000000000000520 37363821 10.1097/HEP.0000000000000520 PMC10653297

[2] Younossi ZM, Golabi P, Paik JM, Henry A, Van Dongen C, Henry L (2023) The global epidemiology of nonalcoholic fatty liver disease (NAFLD) and nonalcoholic steatohepatitis (NASH): a systematic review. Hepatology 77:1335–1347. 10.1097/HEP.0000000000000004 36626630 10.1097/HEP.0000000000000004 PMC10026948

[3] Adam R, Karam V, Cailliez V, O Grady JG, Mirza D, Cherqui D et al (2018) 2018 Annual report of the European liver transplant registry (ELTR)—50-year evolution of liver transplantation. Transpl Int 31:1293–1317. 10.1111/tri.13358 30259574 10.1111/tri.13358

[4] Cotter TG, Charlton M (2020) Nonalcoholic steatohepatitis after liver transplantation. Liver Transpl 26:141–159. 10.1002/lt.25657 31610081 10.1002/lt.25657

[5] Rinella ME, Neuschwander-Tetri BA, Siddiqui MS, Abdelmalek MF, Caldwell S, Barb D et al (2023) AASLD practice guidance on the clinical assessment and management of nonalcoholic fatty liver disease. Hepatology 77:1797–1835. 10.1097/HEP.0000000000000323 36727674 10.1097/HEP.0000000000000323 PMC10735173

[6] European Association for the Study of the Liver, European Association for the Study of Diabetes, European Association for the Study of Obesity (2016) EASL-EASD-EASO clinical practice guidelines for the management of non-alcoholic fatty liver disease. J Hepatol 64:1388–1402. 10.1016/j.jhep.2015.11.004 27062661 10.1016/j.jhep.2015.11.004

[7] Wong VW, Wong GL, Chan RS, Shu SS, Cheung BH, Li LS et al (2018) Beneficial effects of lifestyle intervention in non-obese patients with non-alcoholic fatty liver disease. J Hepatol 69:1349–1356. 10.1016/j.jhep.2018.08.011 30142427 10.1016/j.jhep.2018.08.011

[8] Gonzalez JS, Peyrot M, McCarl LA, Collins EM, Serpa L, Mimiaga MJ et al (2008) Depression and diabetes treatment nonadherence: a meta-analysis. Diabetes Care 31:2398–2403. 10.2337/dc08-1341 19033420 10.2337/dc08-1341 PMC2584202

[9] Goossens L, Braet C, Van Vlierberghe L, Mels S (2009) Loss of control over eating in overweight youngsters: the role of anxiety, depression and emotional eating. Eur Eat Disord Rev 17:68–78. 10.1002/erv.892 18729132 10.1002/erv.892

[10] Leehr EJ, Krohmer K, Schag K, Dresler T, Zipfel S, Giel KE (2015) Emotion regulation model in binge eating disorder and obesity–a systematic review. Neurosci Biobehav Rev 49:125–134. 10.1016/j.neubiorev.2014.12.008 25530255 10.1016/j.neubiorev.2014.12.008

[11] Witaszek T, Babicki M, Brytek-Matera A, Mastalerz-Migas A, Kujawa K, Kloda K (2023) Maladaptive eating behaviours, generalised anxiety disorder and depression severity: a comparative study between adult women with overweight, obesity, and normal body mass index range. Nutrients. 10.3390/nu16010080 38201910 10.3390/nu16010080 PMC10780963

[12] Lim GY, Tam WW, Lu Y, Ho CS, Zhang MW, Ho RC (2018) Prevalence of depression in the community from 30 Countries between 1994 and 2014. Sci Rep 8:2861. 10.1038/s41598-018-21243-x 29434331 10.1038/s41598-018-21243-x PMC5809481

[13] Ruscio AM, Hallion LS, Lim CCW, Aguilar-Gaxiola S, Al-Hamzawi A, Alonso J et al (2017) Cross-sectional comparison of the epidemiology of DSM-5 generalized anxiety disorder across the globe. JAMA Psychiat 74:465–475. 10.1001/jamapsychiatry.2017.0056 10.1001/jamapsychiatry.2017.0056 PMC5594751 28297020

[14] Xiao J, Lim LKE, Ng CH, Tan DJH, Lim WH, Ho CSH et al (2021) Is fatty liver associated with depression? A meta-analysis and systematic review on the prevalence, risk factors, and outcomes of depression and non-alcoholic fatty liver disease. Front Med (Lausanne) 8:691696. 10.3389/fmed.2021.691696 34277666 10.3389/fmed.2021.691696 PMC8278401

[15] Goulart AC, Bianchi LLT, Bismarchi D, Miname MH, Lourencao ACM, Henares BB et al (2023) Sex differences in the relationship between hepatic steatosis, mood and anxiety disorders. J Psychosom Res 168:111216. 10.1016/j.jpsychores.2023.111216 36913766 10.1016/j.jpsychores.2023.111216

[16] Weinstein AA, De Avila L, Kannan S, Paik JM, Golabi P, Gerber LH et al (2022) Interrelationship between physical activity and depression in nonalcoholic fatty liver disease. World J Hepatol 14:612–622. 10.4254/wjh.v14.i3.612 35582293 10.4254/wjh.v14.i3.612 PMC9055201

[17] Tomeno W, Kawashima K, Yoneda M, Saito S, Ogawa Y, Honda Y et al (2015) Non-alcoholic fatty liver disease comorbid with major depressive disorder: the pathological features and poor therapeutic efficacy. J Gastroenterol Hepatol 30:1009–1014. 10.1111/jgh.12897 25619308 10.1111/jgh.12897

[18] Ng CH, Xiao J, Chew NWS, Chin YH, Chan KE, Quek J et al (2022) Depression in non-alcoholic fatty liver disease is associated with an increased risk of complications and mortality. Front Med (Lausanne) 9:985803. 10.3389/fmed.2022.985803 36275825 10.3389/fmed.2022.985803 PMC9582593

[19] Sayiner M, Arshad T, Golabi P, Paik J, Farhat F, Younossi ZM (2020) Extrahepatic manifestations and healthcare expenditures of non-alcoholic fatty liver disease in the Medicare population. Hepatol Int 14:556–566. 10.1007/s12072-020-10038-w 32300995 10.1007/s12072-020-10038-w

[20] Brodosi L, Stecchi M, Marchignoli F, Lucia E, Magnani L, Guarneri V et al (2023) Risk of binge eating disorder in patients with metabolic dysfunction-associated steatotic liver disease. Eat Weight Disord 28:100. 10.1007/s40519-023-01628-2 38055131 10.1007/s40519-023-01628-2 PMC10700210

[21] Alberti KG, Zimmet P, Shaw J (2005) The metabolic syndrome–a new worldwide definition. Lancet 366:1059–1062. 10.1016/S0140-6736(05)67402-8 16182882 10.1016/S0140-6736(05)67402-8

[22] Prati D, Taioli E, Zanella A, Della Torre E, Butelli S, Del Vecchio E et al (2002) Updated definitions of healthy ranges for serum alanine aminotransferase levels. Ann Intern Med 137:1–10. 10.7326/0003-4819-137-1-200207020-00006 12093239 10.7326/0003-4819-137-1-200207020-00006

[23] Bedogni G, Bellentani S, Miglioli L, Masutti F, Passalacqua M, Castiglione A et al (2006) The fatty liver index: a simple and accurate predictor of hepatic steatosis in the general population. BMC Gastroenterol 6:33. 10.1186/1471-230X-6-33 17081293 10.1186/1471-230X-6-33 PMC1636651

[24] Vallet-Pichard A, Mallet V, Nalpas B, Verkarre V, Nalpas A, Dhalluin-Venier V et al (2007) FIB-4: an inexpensive and accurate marker of fibrosis in HCV infection. comparison with liver biopsy and fibrotest. Hepatology 46:32–36. 10.1002/hep.21669 17567829 10.1002/hep.21669

[25] European Association for the Study of the Liver, Asociacion Latinoamericana para el Estudio del Higado (2015) EASL-ALEH clinical practice guidelines: non-invasive tests for evaluation of liver disease severity and prognosis. J Hepatol 63:237–264. 10.1016/j.jhep.2015.04.006 25911335 10.1016/j.jhep.2015.04.006

[26] Eddowes PJ, Sasso M, Allison M, Tsochatzis E, Anstee QM, Sheridan D et al (2019) Accuracy of FibroScan controlled attenuation parameter and liver stiffness measurement in assessing steatosis and fibrosis in patients with nonalcoholic fatty liver disease. Gastroenterology 156:1717–1730. 10.1053/j.gastro.2019.01.042 30689971 10.1053/j.gastro.2019.01.042

[27] Beck AT, Steer RA, Carbin MG (1988) Psychometric properties of the beck depression inventory: twenty-five years of evaluation. Clin Psychol Rev 8:77–100 10.1016/0272-7358(88)90050-5

[28] Martinsen EW, Friis S, Hoffart A (1995) Assessment of depression: comparison between beck depression inventory and subscales of comprehensive psychopathological rating scale. Acta Psychiatr Scand 92:460–463. 10.1111/j.1600-0447.1995.tb09613.x 8837974 10.1111/j.1600-0447.1995.tb09613.x

[29] Lustman PJ, Clouse RE, Griffith LS, Carney RM, Freedland KE (1997) Screening for depression in diabetes using the beck depression inventory. Psychosom Med 59:24–31. 10.1097/00006842-199701000-00004 9021863 10.1097/00006842-199701000-00004

[30] Baggio A, Ferrari R, Partinico M, Vidotto G, Visentin M (1997) Il Beck Depression Inventory per la valutazione della depressione nel dolore cronico. Il contributo degli “item” somatici. Int J Pain Ther 7:4–11

[31] Spielberger CD, Vagg PR (1984) Psychometric properties of the STAI: a reply to Ramanaiah, Franzen, and Schill. J Pers Assess 48:95–97 6707862 10.1207/s15327752jpa4801_16

[32] Lazzari R, Pancheri P (1980) Questionario di valutazione dell'ansia di stato e di tratto (State-Trait Anxiety Inventory). Firenze: Organizzazioni Speciali

[33] Rossini R, Moscatiello S, Tarrini G, Di Domizio S, Soverini V, Romano A et al (2011) Effects of cognitive-behavioral treatment for weight loss in family members. J Am Diet Assoc 111:1712–1719. 10.1016/j.jada.2011.08.001 22027054 10.1016/j.jada.2011.08.001

[34] Tarrini G, Di Domizio S, Rossini R, Romano A, Cerrelli F, Marchesini G et al (2006) Quanto mangio veramente? G Ital Diabetol Metab 26:48–53

[35] Yu M, Shi Y, Gu L, Wang W (2022) “Jolly fat” or “sad fat”: a systematic review and meta-analysis of the association between obesity and depression among community-dwelling older adults. Aging Ment Health 26:13–25. 10.1080/13607863.2020.1857687 33300393 10.1080/13607863.2020.1857687

[36] Gallagher C, Waidyatillake N, Pirkis J, Lambert K, Cassim R, Dharmage S et al (2023) The effects of weight change from childhood to adulthood on depression and anxiety risk in adulthood: a systematic review. Obes Rev 24:e13566. 10.1111/obr.13566 37062534 10.1111/obr.13566

[37] Choi JM, Chung GE, Kang SJ, Kwak MS, Yang JI, Park B et al (2020) Association between anxiety and depression and nonalcoholic fatty liver disease. Front Med (Lausanne) 7:585618. 10.3389/fmed.2020.585618 33537324 10.3389/fmed.2020.585618 PMC7848018

[38] Kim D, Dennis BB, Cholankeril G, Ahmed A (2023) Association between depression and metabolic dysfunction-associated fatty liver disease/significant fibrosis. J Affect Disord 329:184–191. 10.1016/j.jad.2023.02.101 36841305 10.1016/j.jad.2023.02.101

[39] Labenz C, Huber Y, Michel M, Nagel M, Galle PR, Kostev K et al (2020) Nonalcoholic fatty liver disease Increases the risk of anxiety and depression. Hepatol Commun 4:1293–1301. 10.1002/hep4.1541 32923833 10.1002/hep4.1541 PMC7471420

[40] Nierenberg AA, Agustini B, Kohler-Forsberg O, Cusin C, Katz D, Sylvia LG et al (2023) Diagnosis and treatment of bipolar disorder: a review. JAMA 330:1370–1380. 10.1001/jama.2023.18588 37815563 10.1001/jama.2023.18588

[41] Mansur RB, Brietzke E, McIntyre RS (2015) Is there a “metabolic-mood syndrome”? A review of the relationship between obesity and mood disorders. Neurosci Biobehav Rev 52:89–104. 10.1016/j.neubiorev.2014.12.017 25579847 10.1016/j.neubiorev.2014.12.017

[42] Gill H, Gill B, El-Halabi S, Chen-Li D, Lipsitz O, Rosenblat JD et al (2020) Antidepressant medications and weight change: a narrative review. Obesity (Silver Spring) 28:2064–2072. 10.1002/oby.22969 33022115 10.1002/oby.22969

[43] Estes C, Anstee QM, Arias-Loste MT, Bantel H, Bellentani S, Caballeria J et al (2018) Modeling NAFLD disease burden in China, France, Germany, Italy, Japan, Spain, United Kingdom, and United States for the period 2016–2030. J Hepatol 69:896–904. 10.1016/j.jhep.2018.05.036 29886156 10.1016/j.jhep.2018.05.036

[44] Lazarus JV, Mark HE, Anstee QM, Arab JP, Batterham RL, Castera L et al (2022) Advancing the global public health agenda for NAFLD: a consensus statement. Nat Rev Gastroenterol Hepatol 19:60–78. 10.1038/s41575-021-00523-4 34707258 10.1038/s41575-021-00523-4

[45] Francque SM, Marchesini G, Kautz A, Walmsley M, Dorner R, Lazarus JV et al (2021) Non-alcoholic fatty liver disease: a patient guideline. JHEP Rep 3:100322. 10.1016/j.jhepr.2021.100322 34693236 10.1016/j.jhepr.2021.100322 PMC8514420

[46] Lazarus JV, Mark HE, Allen AM, Arab JP, Carrieri P, Noureddin M et al (2023) A global research priority agenda to advance public health responses to fatty liver disease. J Hepatol 79:618–634. 10.1016/j.jhep.2023.04.035 37353401 10.1016/j.jhep.2023.04.035

[47] Associazione Italiana per lo Studio del Fegato, Societa Italiana di Diabetologia, Societa Italiana dell’Obesità (2022) Non-alcoholic fatty liver disease in adults 2021: a clinical practice guideline of the Italian Association for the Study ofhe Liver (AISF), the Italian Society of Diabetology (SID) and the Italian Society of Obesity (SIO). Nutr Metab Cardiovasc Dis 32:1–16. 10.1016/j.numecd.2021.04.028 34924246 10.1016/j.numecd.2021.04.028

[48] Lazarus JV, Palayew A, Carrieri P, Ekstedt M, Marchesini G, Novak K et al (2021) European ‘NAFLD Preparedness Index’—Is Europe ready to meet the challenge of fatty liver disease? JHEP Rep 3:100234. 10.1016/j.jhepr.2021.100234 33733078 10.1016/j.jhepr.2021.100234 PMC7937562

